# Speciation in *Thaparocleidus* (Monogenea: Dactylogyridae) Parasitizing Asian Pangasiid Catfishes

**DOI:** 10.1155/2013/353956

**Published:** 2013-11-20

**Authors:** Andrea Šimková, Celine Serbielle, Antoine Pariselle, Maarten P. M. Vanhove, Serge Morand

**Affiliations:** ^1^Department of Botany and Zoology, Faculty of Science, Masaryk University, Kotlářská 2, 611 37 Brno, Czech Republic; ^2^Institut de Recherche sur la Biologie de l'Insecte, Faculté des Sciences et Techniques, UMR CNRS 6035, 37200 Tours, France; ^3^Institut des Sciences de l'Evolution, IRD-CNRS-UM2, Université Montpellier 2, CC065, 34095 Montpellier Cedex 05, France; ^4^Laboratory of Biodiversity and Evolutionary Genomics, University of Leuven, Charles Deberiotstraat 32, 3000 Leuven, Belgium

## Abstract

The phylogeny of monogeneans of the genus *Thaparocleidus* that parasitize the gills of Pangasiidae in Borneo and Sumatra was inferred from molecular data to investigate parasite speciation. The phylogeny of the Pangasiidae was also reconstructed in order to investigate host-parasite coevolutionary history. The monophyly of *Thaparocleidus* parasitizing Pangasiidae was confirmed. Low intraspecies molecular variability was observed in three *Thaparocleidus* species collected from geographically distant localities. However, a high intraspecies molecular variability was observed in two *Thaparocleidus* species suggesting that these species represent a complex of species highly similar in morphology. Distance-based and tree-based methods revealed a significant global fit between parasite and host phylogenies. Parasite duplication (i.e., intrahost speciation) was recognized as the most common event in *Thaparocleidus*, while the numbers of cospeciation and host switches were lower and similar to each other. When collapsing nodes correspond to duplication cases, our results suggest host switches in the *Thaparocleidus*-Pangasiidae system precluding congruence between host and parasite trees. We found that the morphometric variability of the parasite attachment organ is not linked to phylogeny, suggesting that the attachment organ is under adaptive constraint. We showed that haptor morphometry is linked to host specificity, whereby nonspecific parasites display higher morphometric variability than specialists.

## 1. Introduction

The speciation of free-living organisms is thought to be caused by two main mechanisms: allopatric speciation, which results from reproductive isolation due to extrinsic factors such as geographical barriers [1], and nonallopatric speciation such as sympatric speciation, which requires intrinsic barriers for reproductive isolation [2, 3]. In parasites, speciation is usually linked to the evolutionary history of their host species, with host speciation inducing parasite speciation when each incipient host species has inherited parasite populations that subsequently diverge from a common ancestor [4]. Therefore, the allopatric speciation of parasites may occur when extrinsic barriers prevent parasite reproduction among isolated host populations. For example, this can occur when the host species are geographically isolated. On the other hand, sympatric speciation (geographic sympatry, within host sympatry or within microhabitat sympatry) can occur when the isolation of parasite populations is maintained by intrinsic barriers [5] and is therefore independent of host speciation events. Sympatric speciation could explain a large part of parasite diversity [4]. According to Kunz [6], sympatric speciation is more likely to occur in parasites than in free-living organisms, considering that the isolation of parasite populations seems to be accomplished more easily than in free-living organisms. The isolation processes and intrinsic barriers among parasite populations, such as host choice when the parasite shows a local host preference or mate choice when mating between two parasites is impossible, most likely will lead to parasite sympatric speciation [5]. Parasite sympatric speciation may occur within a single host species; that is, a parasite lineage has evolved within a single host species without any isolation of host populations. The key assumption here is that congeneric parasite species on the same host are sister species and that their occurrence is the result of one or more events of intrahost speciation [7].

Cophylogenetic studies comparing the evolutionary histories of parasites and their associated hosts may help us to further explore parasite speciation mechanisms [8]. Congruence of host and parasite phylogenies is considered evidence of cospeciation, that is, the concurrent speciation of both associated partners [8]. However, congruent trees are not always linked to cospeciation (see [9]). Incongruent phylogenies are often explained by host-switching events or parasite duplication. However, testing for cospeciation or codivergence (i.e., simultaneous speciation or divergence of host and parasite lineages, while cospeciation is a special kind of codivergence in which the end products of the divergence process are considered separate species) requires the combination of distance-based, tree-based, and data-based (these methods are used to determine the cause of topological incongruence between host and parasite trees) cophylogenetic methods [10]. Indeed, discriminating between trees that are concordant as a result of codivergence and trees that are concordant for reasons unrelated to codivergence necessitates the comparison of topological similarities between not only host and parasite trees but also timing of events [10].

Speciation in parasites has been mostly explained by their life-history traits, such as host specificity. Generally, a parasite living on/in one host species is considered a specialist, and a parasite living on/in at least two species is considered a generalist [11]. Brooks and McLennan [4] hypothesized that the chance of colonizing new host species, that is, host switching, and the subsequent speciation are inversely related to the degree of host specificity, which supposes that cospeciation and intrahost speciation are more frequent in parasites having a narrow host range. For example, in the highly host specific chewing lice parasitizing pocket gophers, cospeciation was found to be the main speciation event [12, 13]. 

Monogeneans, a group of mostly ectoparasitic flatworms predominantly found in fishes, seem to be an ideal model for investigating parasite diversification for at least three reasons. First, monogeneans are a highly diverse parasite group in terms of species richness [14]. Second, many monogenean species tend to be host specific, that is, infecting only one or a few host species [15], and also niche specific, that is, restricted to a particular habitat within the host species [7, 16]. Third, monogeneans are parasites with a direct life cycle (only one host species is involved in their life cycle), which may simplify the analyses of host-parasite associations compared to endoparasites with a complex life cycle (including intermediate and definitive host species throughout various stages of the life cycle). To date, several studies have investigated the speciation and diversification of different congeneric monogenean species. These studies do not show strong patterns of cospeciation, despite the high host specificity of monogenean parasites, but they suggest that monogeneans mostly diversify either through host switching [17–20] or by intrahost speciation [7, 21]. Host specificity, varying between the different monogenean models investigated, is considered to be an important parasite trait involved in monogenean speciation processes. 

Monogeneans possess a posterior attachment organ, called a haptor, which is supposed to be linked to both specialization and adaptation [22]. Morand et al. [23] hypothesized that a link between morphological and phylogenetic distances may reflect a nonadaptive trend due to a high phylogenetic inertia, with sister species possessing similar haptors because they have inherited them from a common ancestor. Conversely, a link between the morphometrics of the monogenean haptor and host specificity may reveal a potential adaptation. Indeed, a higher variability of the attachment organ was shown in generalists compared to specialists in two groups of monogenean species [24, 25]. 

*Thaparocleidus *(Dactylogyridae, Ancylodiscoidinae) are gill monogeneans in siluriform fishes [26]. Nonmonophyly of *Thaparocleidus* was shown by Wu et al. [27]. To date, 43 *Thaparocleidus* species have been described from 18 species of Pangasiidae ([28] and references therein). These monogenean species are highly host specific: most of them are restricted to one host species. Pangasiid fishes are distributed in the main rivers and estuaries of South East Asia and occasionally in the sea [29]. The speciation processes involved in their diversification seem to be closely related to tectonic events that have formed the current river network in South East Asia as well as to sea level changes [30, 31]. Within this historical-biogeographical framework, we hypothesized that the speciation and diversification of specific *Thaparocleidus* should be closely related to the processes of Pangasiidae diversification. Moreover, we want to compare the diversification of *Thaparocleidus *species infecting the same host species to the processes observed in another well-studied freshwater dactylogyrid monogenean, *Dactylogyrus*. We hypothesize that, similar to *Dactylogyrus *parasitizing cyprinid fishes [7], intrahost speciation is an important speciation mechanism for *Thaparocleidus*. 

The aim of this study was to use molecular phylogenetic reconstruction of *Thaparocleidus *parasitizing Pangasiidae in combination with cophylogenetic analyses to investigate speciation and diversification in this monogenean genus. We also investigated whether the morphometry of the attachment organ is phylogenetically constrained and linked to host specificity. 

## 2. Materials and Methods

### 2.1. Parasite Sampling

Monogeneans identified as belonging to 16 different *Thaparocleidus* species were collected from freshwater fish species in the Indonesian islands Borneo and Sumatra (South East Asia). The fish were bought from the market or directly from fishermen and identified following Roberts and Vidthayanon [29]. After dissection, their left gill arches were preserved in 70% ethanol. Species identification was based on the morphology of sclerotized parts of the attachment and reproductive organs following Lim [32] and Pariselle et al. [28, 33–36]. All *Thaparocleidus* species collected for this study were found on a single host species except for *T. caecus*, which was collected from *Pangasius nasutus* and *Pangasianodon hypophthalmus* (this host species has been introduced over the entire region of Southeast Asia for aquaculture purposes). However, host specificity in this study (Table 1) was evaluated at global level; that is, the data on host range of each analyzed *Thaparocleidus* species were retrieved from published studies [28, 33–37]. Thus, parasite species were separated into two categories: specialist parasitizing a single host species and generalist parasitizing at least two different host species [38]. The geographical distribution of all fish species collected for this study was limited to Indonesia except for *Pangasius micronema*, which is distributed widely in South East Asia. The localities of the collected fish are given in Table 1.

### 2.2. DNA Extraction, Amplification, and Sequencing

DNA extraction was performed by the chelex method. Each monogenean specimen was disrupted in a 5% chelex solution with proteinase K (0.12 mg/mL). Partial 18S rDNA and entire ITS1 region were amplified in one round using the primers S1 (5′- ATTCCGATAACGAACGAGACT-3′) [39] and IR8 (5′-GCTAGCTGCGTTCTTCATCGA-3′) that anneal to the 18S and 5.8S rDNA genes, respectively [40]. Each amplification reaction was performed in a final volume of 25 *μ*L containing 1.5 units of *Taq* polymerase, 1X buffer containing MgCl_2_, 2.5 mM of each dNTP, 0.4 *μ*M of each primer, and 5 *μ*L of DNA. PCR was carried out using the following steps: 4 min at 95°C followed by 40 cycles of 1 min at 92°C, 1 min at 55°C, and 1 min 30 s at 72°C and 10 min of final elongation at 72°C. The PCR products were checked in a 1% agarose gel. The PCR products were purified by a ExoSAP-IT kit (USB) and were directly sequenced using the PCR primers. Sequencing was carried out using an ABI Prism BigDye Terminator Cycle Sequencing kit (Applied Biosystems) and electrophoresis was performed on an automated sequencer (MegaBace 500). New sequences were deposited in GenBank (see Table 1 for the accession numbers).

### 2.3. Phylogenetic Analyses

DNA sequences were aligned using ClustalW multiple alignment [41] in BioEdit Sequence Alignment Editor v.7.0.9 [42]. As the alignment for these closely related species was straightforward, gaps were included in the sequence alignment subjected to the phylogenetic analyses. Firstly, phylogenetic analyses were performed using partial 18S rDNA sequences, to allow inclusion of outgroups, in order to be able to subsequently root *Thaparocleidus* tree reconstructions. Four species, belonging to Ancylodiscoidinae and infecting nonpangasiid siluriforms, were used as outgroup to root the phylogeny of *Thaparocleidus* species parasitizing Pangasiidae: *Thaparocleidus siluri* (AJ490164), *T. vistulensis* (AJ490165) (both of them are monogenean parasites of the European silurid catfish species *Silurus glanis*), and two monogenean species from catfishes collected in West Africa, *Schilbetrema* sp. (HG491495) from the schilbeid catfish *Schilbe intermedius* and *Quadriancanthus* sp. (HG491496) from the airbreathing clariid *Heterobranchus bidorsalis*. As mentioned above, *Thaparocleidus* is not monophyletic; that is, *Thaparocleidus *parasitizing Siluridae and* Thaparocleidus *parasitizing Pangasiidae comprise different clades. Therefore, Wu et al. [27] proposed taxonomic revision of the species recently included in *Thaparocleidus*. Hence, *T. siluri *and *T. vistulensis *may be included as outgroup taxa. Subsequent phylogenetic analyses were performed using a concatenated dataset of partial 18S rDNA and ITS1 including only sequences of *Thaparocleidus* species parasitizing Pangasiidae. Intraspecific variability was explored using uncorrected p-distances (i.e., calculating the proportions of different nucleotide sites).

Phylogenetic analyses using minimum evolution (ME), maximum parsimony (MP), and maximum likelihood (ML) were performed in PAUP∗4b10 [43]. The Bayesian analyses were conducted using MrBayes 3.1 [44]. ModelTest [45] was used to select the optimal evolutionary model for each dataset, based on hierarchical likelihood ratio tests. The selected model was applied in the ME, ML, and BI tree reconstructions. ME analyses were performed using a heuristic search with a distance optimality criterion [46]. The search for the best ML tree was done via a heuristic search using the tree bisection reconnection branch-swapping algorithm (TBR). MP analyses were performed using a heuristic search algorithm with a stepwise random addition sequence running on unweighted informative characters and TBR branch swapping. The degree of “tree-likeness” in data under a parsimony model for character change was measured by consistency index (CI) and retention index (RI). Sequence alignment and trees were deposited to TreeBase, a database of phylogenetic knowledge, and are available at http://purl.org/phylo/treebase/phylows/study/TB2:S14752. Support values for internal nodes were estimated using a bootstrap resampling procedure with 1000 replicates [47]. BI analyses were performed using four Monte Carlo Markov Chains (MCMC) running on a given number of generations (starting at 10,000 and increasing until standard deviation was below 0.01), with trees being sampled every 100 generations. Log likelihoods of the saved trees were graphically inspected, and all trees before stationary were discarded as “burn-in.” Two replicates were conducted for the Bayesian MCMC runs. The posterior probabilities for internal nodes were determined for all trees left in the plateau phase with the best likelihood scores. In accordance with Wahlberg et al. [48] and Yang et al. [49], clade support in phylogenetic trees indicated by bootstrap values (BP) or posterior probabilities (PP) was considered as follows: weak support 50–63%/0.5–0.69, moderate support 64–75%/0.7–0.84, good support 76–88%/0.85–0.94, and strong support 89–100%/0.95–1.00. 

The sequences of cytochrome *b* obtained from Pouyaud et al. [30] were used for the phylogenetic reconstruction of Pangasiidae investigated in our study. Two Asian siluriforms, *Laides hexanema* and *Pseudeutropius brachypopterus*, belonging to Schilbeidae were used as outgroup [30]. Nucleotide sequences were aligned and then translated to amino acid sequences in MEGA v. 3.1 [50]. The best appropriate model of protein evolution was selected in ProtTest v. 2.4 [51] using the Akaike information criterion. The trees were reconstructed using both fast and slow strategies. Using the fast strategy, the tree with BIONJ topology and branch length optimized under the best-fit model was obtained. Using the slow strategy, the best ML tree with both branch length and topology optimized under the best-fit model was obtained. A nonparametric bootstrap was calculated in PhyML 3.0 using 1000 replicates [52].

### 2.4. Cophylogenetic Analyses

Two methods of coevolutionary analyses were applied: a distance-based method called ParaFit [53] implemented in CopyCat [54] and a tree-based method implemented in Jane 4.0 [55]. A tanglegram representing the host-parasite associations was reconstructed in TreeMap 1.0 [56]. 

Distance-based methods use host and parasite distance matrices and host-parasite associations to determine whether hosts and parasites are randomly associated. The global fit between host and parasite trees is computed and tested by randomizing individual host-parasite associations. ParaFit was also used to test whether a particular host-parasite association contributed to this global fit. The permutational tests of significance were calculated using 999 permutations. This method was shown to be useful for studying host-parasite cophylogeny using congeneric monogeneans parasitizing fish [17, 21]. 

Tree-based methods use tree topologies to assess the fit between host and parasite phylogenies. These methods are aimed at the reconstruction of shared evolutionary history between hosts and parasites with the smallest number of hypothesized historical events (this is expressed by “cost”). Each event has an attributed cost and the reconstruction with the lowest global cost is searched. Jane supports multihost parasites and multiparasite hosts. In addition, Jane 4 supports polytomies. In our study, we applied seven models with different event cost schemes. TreeMap and TreeFitter [57] models classically used four types of coevolutionary events, that is, cospeciation, duplication, host switching, and sorting event; however, Jane applies a fifth type of coevolutionary events called “failure to diverge” (host speciation is not followed by parasite speciation, and the same parasite species occur on the new host species). The cost for “failure to diverge” was added in TreeMap and TreeFitter models following Mendlová et al. [21]. The cophylogenetic analyses were performed using the following genetic parameters: 1000 generations and 100 as a population size. Statistical tests were computed using 999 randomizations using the method of random parasite tree. The *Thaparocleidus* tree inferred from the analysis of combined 18S rDNA and ITS1 data and the Pangasiidae tree inferred from cytochrome *b* were used in the cophylogenetic analyses. 

### 2.5. Parasite Morphometry

**Figure 1:**
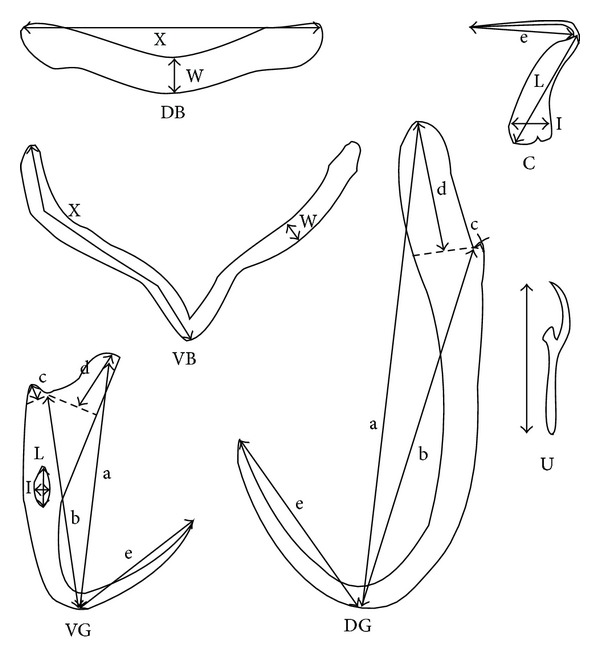
Measurements of the sclerotized part of haptor in *Thaparocleidus* (DB: dorsal transversal bar, VB: ventral transversal bar, VG: ventral gripus, DG: dorsal gripus, C: cuneus, and U: uncinulus, following [28]).

A total of 9 specialist species and 6 generalist species sampled in this study and identified on the basis of morphology were measured (morphometric data for *T. citreum* were not available; see Table 1). Morphometric measurements were taken from 10 individuals from each parasite species. 20 variables describing the haptor were taken into account for statistical analyses. The variables used are represented in Figure 1. The Mahalanobis distances between species were calculated for haptor morphometrics. Phylogenetic distances expressed as patristic distances were calculated between species in PAUP∗4b10 [43]. The existence of a correlation between morphometric distances and phylogenetic distances was tested using a Mantel test. A significant correlation indicated phylogenetic inertia.

Next, we performed a principal component analysis (PCA) on the measurements of the haptor. We extracted the first two principal components as they represented 55.6% and 13.4% of the total variance. We tested the effect of host specificity on parasite haptor morphometry by performing a nonparametric Wald-Wolfowitz test on the values of the first two axes between the generalists and specialists.

## 3. Results

### 3.1. Intraspecies Variability

Within-species variability was compared between individuals collected from different host species (*T. caecus* 1 from *Pangasius nasutus* and *T. caecus* 2 from *Pangasianodon hypophthalmus*). Pairwise comparisons showed a 10.07% variability in combined partial 18S and ITS1 sequences. When comparing conspecific *Thaparocleidus* individuals parasitizing the same host species but collected from different islands (Borneo and Sumatra), pairwise comparisons revealed a 0.31% variability between *T. durandi* 1 (from *Pangasius micronema* collected in Borneo) and *T. durandi* 2 (from *Pangasius micronema* collected in Sumatra). Pairwise comparisons between individuals parasitizing the host species but collected from different rivers in Sumatra revealed a 0.62% variability between *T. rukyanii* 1 collected from Musi River and *T. rukyanii* 2 collected from Batang Hari River and a 0.30% variability between *T. summagracili*s 1 from Musi River and *T. summagracilis* 2 from Batang Hari River. A variability of 5.79% was recorded between *T. alatus* 1 collected from Musi River and *T. alatus* 2 collected from Batang Hari River.

### 3.2. Phylogenetic Analyses Using 18S rDNA

All of the presented phylogenetic analyses show that two individuals of *T. alatus* found on *P. nasutus* from different isolated localities occupy different positions in the phylogenetic trees. Similarly, two individuals morphologically identified as *T. caecus* but parasitizing different host species, *Pangasius nasutus* and *Pangasianodon hypophthalmus*, both collected in Borneo, did not represent sister species. Rather, these species were included in different well-supported clades. 

**Figure 2:**
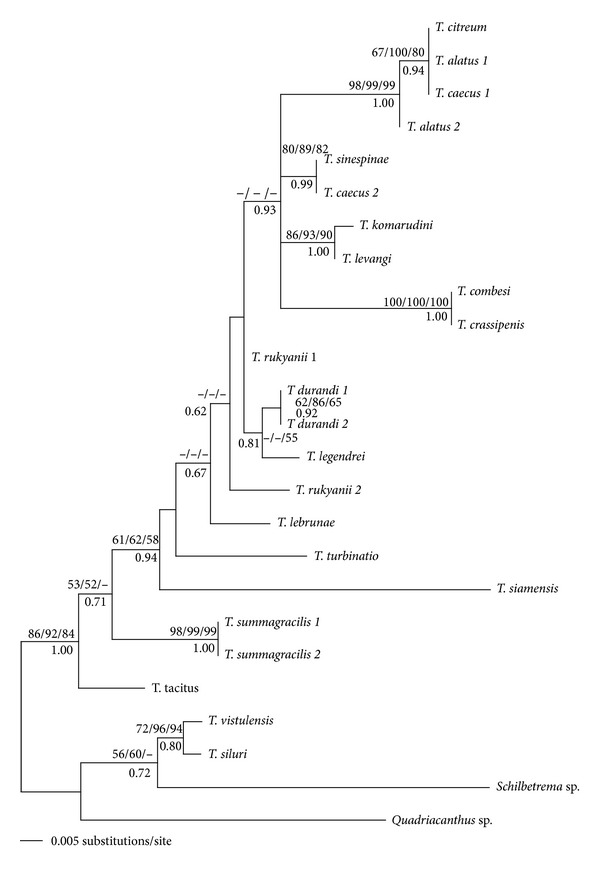
ML tree inferred from the analyses of partial 18S rDNA sequences of species belonging to Ancylodiscoidinae. Numbers above branches indicate bootstrap values resulting from ML/MP/ME analyses; numbers below branches indicate posterior probabilities resulting from BI analysis.

The *Thaparocleidus* species parasitizing fish species of Pangasiidae and four other species of Ancylodiscoidinae were included in the first phylogenetic analyses (see Section 2). The sequence alignment was comprised of 393 unambiguously alignable positions (including 7 positions with gaps), of which 80 were variable and 45 were parsimony informative. The K80+G model was selected as the best appropriate model by ModelTest, and the heterogeneity and substitution rate were approximated by a gamma distribution with shape parameter *α* = 0.2165 and substitution rate matrix: A-C = 1.0000, A-G = 6.2288, A-T = 2.5937, C-G = 2.5937, C-T = 6.2288, and G-T = 1.0000. The MP analysis provided 54 equally parsimonious trees with 120 steps (CI = 0.758, RI = 0.803). The BI analysis was conducted by running MCMC for 300,000 generations. All phylogenetic analyses yielded a similar tree topology (the ML tree is shown in Figure 2 including BP for ML, MP, ME, and PP for BI analyses). Several terminal clades including *Thaparocleidus* of Pangasiidae with moderate, good, or strong support based on ML, MP, and ME analyses and good or strong support based on BI analyses were recognized from the phylogenetic reconstruction using 18S rDNA data. However, many phylogenetic relationships displayed low resolution or were unresolved using ML, MP, and ME analyses, and some of them were resolved only under BI (PP from 0.62 to 0.93). However, the fact that the values of PP tend to be higher than of BP is well known [58]. *Thaparocleidus* of Pangasiidae form a monophyletic group. 

### 3.3. Phylogenetic Analyses Using Combined Data 18S rDNA and ITS1

The congruence of 18S and ITS1 data sets was tested using the partition homogeneity test implemented in PAUP∗4b10 [43]. No significant difference was found between 18S rDNA and ITS1 (*P* = 0.084). Therefore, the next analyses were performed using the concatenated dataset, including 21 sequences of *Thaparocleidus* parasites of Pangasiidae. The phylogenetic trees were oriented using the results obtained from the 18S rDNA analyses. 

**Figure 3:**
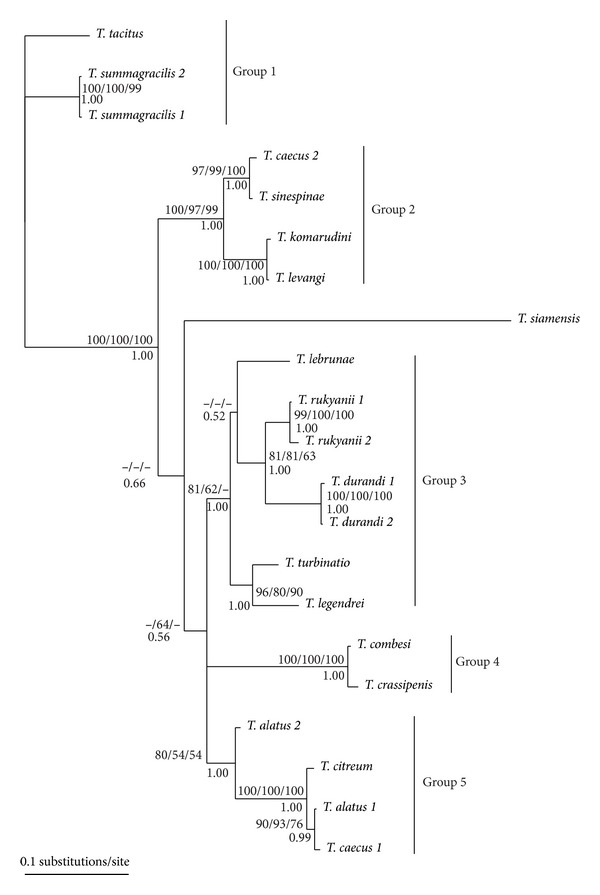
Bayesian topology for *Thaparocleidus* species of Pangasiidae based on combined data of partial 18S rDNA and ITS1 region. Numbers below branches indicate posterior probabilities resulting from BI analysis; numbers above branches indicate bootstrap values resulting from ML/MP/ME analyses.

The sequence alignment was comprised of 666 unambiguously alignable positions (including 36 positions with gaps), of which 232 were variable and 172 were parsimony informative. The TVM+I+G model was selected by ModelTest including equal frequencies of nucleotide bases, with a proportion of invariable sites pi = 0.3871, and the heterogeneity and substitution rate were approximated by a gamma distribution with shape parameter *α* = 0.6119, with the following substitution rate matrix: A-C = 0.8437, A-G = 5.6868, A-T = 3.0736, C-G = 0.8639, C-T = 5.6868, and G-T = 1.0000. The consensus tree obtained from the BI analysis is shown in Figure 3; the values of BP for ML, ME and MP analyses and PP for the BI analysis are included in this figure. The MP analysis provided 2 equally parsimonious trees with 493 steps (CI = 0.659, RI = 0.761). On the basis of the 18S rDNA analyses, *Thaparocleidus tacitus*, a parasite of *Pangasius micronema*, was used for rooting. All phylogenetic analyses yielded a similar tree topology; that is, five clades were recovered in all reconstructions (Figure 3). Three clades (group 1, group 2, and group 4) were strongly supported by BP or PP in all analyses, and two of them were weakly supported by MP (group 3) or by MP and ME (group 5), well supported by ML, and strongly supported by BI analyses (group 3 and group 5). The position of *T. siamensis* was slightly variable using different methods, but this species was always included in a large strongly supported clade of *Thaparocleidus* species including group 2, group 3, group 4, and group 5 (Figure 3). The different position of *T. siamensis* in phylogenetic trees likely results from long branch attraction in the MP analysis and long branch repulsion in the ML analysis [59]. However, no effect of *T. siamensis* on the general topology of Asian *Thaparocleidus* was recognized; that is, an identical global topology was obtained when either excluding or including *T. siamensis.*

### 3.4. Fish Phylogeny

**Figure 4:**
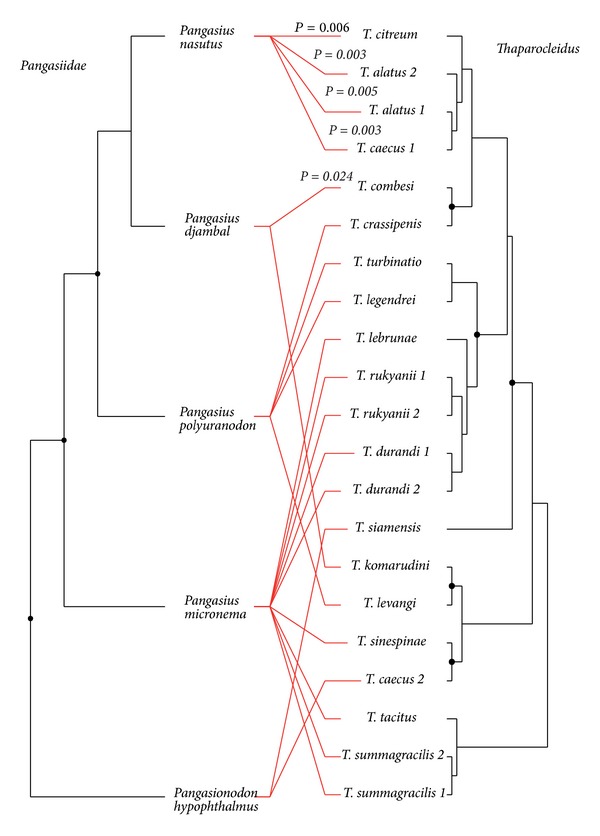
Tanglegram of *Thaparocleidus* and Pangasiidae species deduced from comparison of parasite tree inferred from combined data of 18S rDNA and ITS1 sequences and the fish tree obtained from cytochrome *b* analyses. *P*-values resulting from Parafit for significant host-parasite links are included.

The sequence alignment of cytochrome *b* was comprised of 539 unambiguously alignable positions, of which 197 were variable and 150 were parsimony informative. The amino acids alignment was comprised of 179 amino acids. MtMam+I was selected as the best appropriate model of protein evolution using both the slow and fast strategies in ProtTest with a proportion of invariable sites, pi = 0.822. The topology of the best ML tree is included in Figure 4. The phylogenetic relationships between the analyzed species of Pangasiidae were well resolved (all BP > 80) using both fast (BIONJ) and slow (ML) analyses and including *Laides hexanema* and *Pseudeutropius brachypopterus* as outgroup taxa. *Pangasionodon hypophthalmus* has a basal position to the group of the four *Pangasius* species that were analyzed. 

### 3.5. Host-Parasite Associations

A tanglegram of the *Thaparocleidus* parasite species and their Pangasiidae fish hosts is shown in Figure 4. For *Thaparocleidus* species, the fully resolved ME topology was included (this tree is required to use TreeMap). Using ParaFit running in CopyCat, the overall cophylogenetic structure was highly significant (*P* = 0.013). The test computed for individual host-parasite links showed that five links out of 21 contributed significantly (*P* < 0.05) to this global fit (Figure 4). 

We explored seven models with different event cost schemes (Table 2) previously applied for cophylogenetic studies using Jane, TreeMap, or TreeFitter programs. The analyses revealed a significant global structure using all models except for one, assigning a higher cost to duplication than to other events (Table 2). The number of cospeciations and host switching events (always considered subsequent to duplication in Jane) was similar under all models except the codivergence-adjusted model, which assigned the same cost to cospeciation and host switch and no cost for duplication. Under all event cost settings, cophylogenetic reconciliation revealed that duplication (i.e., parasite speciation without corresponding host speciation) was the most frequent coevolutionary event (see Table 2). The tanglegram of *Thaparocleidus*-Pangasiidae associations demonstrated that intrahost duplication was mostly found in *P. micronema*, the fish species in which the highest diversity of *Thaparocleidus* species was recorded (see Table 1). However, as shown in this tanglegram, intrahost duplications were also reported in *P. nasutus* and *P. polyuranodon*. When the intrahost duplications were removed from reconstruction (i.e., after collapsing nodes in the parasite tree that correspond to duplications), no significant global structure (*P* > 0.05) was found using any of the models. The same number of cospeciation and host switch was inferred as in the analyses using all *Thaparocleidus* species. 

### 3.6. Morphometric Variability of the Attachment Organ

**Figure 5:**
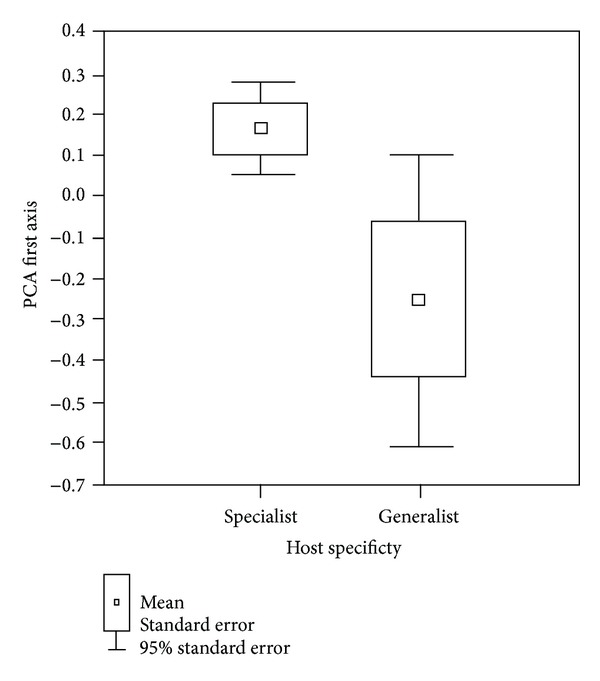
Morphometric variability of the parasite attachment organ estimated by the first axis of principal component analysis. The nonspecific parasites (i.e., generalists) differ significantly from specific parasites. The generalists show significant higher variance in haptor morphometry than specific parasites.

No significant correlation between the Mahalanobis distances of the haptor and the patristic phylogenetic distances calculated between pairs of monogenean species was found (Mantel test, *F*_1,105_ = 8.764; *R* ^2^ = 0.08, *P* = 0.14). We performed a PCA on 20 morphometric variables of the haptor. The first two principal component axes were extracted (eigenvalue of the first axis = 11.12 with 55.6% of the total variance; the eigenvalue of the second axis = 2.68 with 13.40% of the total variance). We compared the values of the first two axes, PCA1 and PCA2, with host specificity (specific versus nonspecific) as a factor. Significant difference in haptor morphometry was found between the specific and nonspecific parasites using a Wald-Wolfowitz test (*Z* = −6.064, *P* < 0.001 for PC1 and *Z* = −5.548, *P* < 0.001 for PC2). Moreover, the nonspecific parasite species varied more in their morphometrics than the specific ones (*F* test, *P* < 0.05) (Figure 5).

## 4. Discussion

### 4.1. *Thaparocleidus* Species: Morphological versus Molecular Species Concept

In monogeneans, species identification is generally based on the morphology of two sclerotized organs, the attachment organ (the haptor) and the reproductive organ, including the copulatory piece and the vagina [60]. The morphology of the haptor is considered useful for parasite determination at the genus level, while the reproductive organ is more suitable for identification at the species level, probably because of its higher rate of change [27, 61, 62]. However, in some cases, identification is problematic at the generic as well as the specific level. Therefore, molecular identification is a helpful tool in resolving taxonomic problems in cases where the morphological “boundaries” among monogenean genera or species groups are ambiguous [27, 61, 62]. 

In our study, molecular phylogeny confirmed the monophyly of *Thaparocleidus* species parasitizing pangasiid hosts from Borneo and Sumatra. Wu et al. [27] showed that the species recently included in *Thaparocleidus* do not constitute a monophyletic group; that is, *Thaparocleidus* parasitizing Siluridae and *Thaparocleidus* parasitizing Pangasiidae form two divergent genetic lineages. Our results demonstrate that molecular data are not only helpful in recovering monophyly in monogeneans but also allow the discrimination of allegedly conspecific and morphologically identical species. In our study, intraspecies genetic variability was found between parasite individuals morphologically identified as belonging to the same species, which were collected from geographically distant locations, that is, for *T. summagracilis*, *T. durandi*, and *T. rukyanii*, each of them parasitizing a single host species collected from different Indonesian islands or two different rivers in Sumatra. However, intraspecies variability observed in *T. caecus* parasitizing two different host species and *T. alatus* parasitizing the same host species is very high, suggesting that each of these two species represents a potential complex of morphologically similar species. On the other hand, some morphologically well-identified *Thaparocleidus* species in our study showed a low level of molecular divergence. Similarly, consistently distinctive species separated by low genetic distances were also recognized within *Ligophorus* (also dactylogyrid gill monogeneans but parasitizing Mugilidae) ([63], Marchiori et al., unpublished). These are likely species complexes explained by recent rapid speciation and diversification. 

The morphology of the male copulatory organ of *Thaparocleidus* was suggested as a key determinant for separating the clades recovered in molecular phylogenetic reconstruction [27]. A similar argument was used for monogeneans belonging to *Cichlidogyrus*, parasitizing Cichlidae, by Wu et al. [62]. However, Pouyaud et al. [61] and Vignon et al. [64] suggested that the morphology of the attachment organ is more suitable to infer phylogenetic relationships among major lineages in these cichlid monogeneans. The association between attachment organ morphology and (molecular based) phylogeny has also been documented for *Dactylogyrus*, a highly specific group of monogeneans of Cyprinidae [65]. However, species sharing the same morphological traits are not necessarily derived from a common ancestor. Moreover, the evolution of sclerotized organs is not neutral and seems to be under adaptive constraints in case of the attachment organ [23, 66]. In our study, the morphological variability of the haptor was not linked to phylogenetic distances, suggesting that the morphological variability of these sclerotized organs is not inherited from a common ancestor and may be under adaptive constraint.

### 4.2. Morphometric and Molecular Variability versus Host Specificity

Interspecific variability in ITS1 sequences and in haptor morphometry was previously shown in generalist monogeneans such as species belonging to *Dactylogyrus* and *Lamellodiscus* [24, 25]. Moreover, *Lamellodiscus* generalists have a higher intraspecies molecular variability and a higher variance of haptor morphometry than do specialists. In our study, we also found that the variance in haptor morphometry is higher in *Thaparocleidus* generalists than in specialists. Kaci-Chaouch et al. [25] proposed two alternative hypotheses to explain why the variance in haptor morphometry is higher in generalists than in specialists. Generalists exhibit a higher variance because (i) they use different host species representing a wide range of niches, which can exert different pressures on morphology, or (ii) they have a higher morphometric variability of the attachment organ which may allow parasites to colonize more host species. Therefore, morphometric variability can have a major impact on parasite speciation processes regardless of host speciation by restricting specialists within a particular host and habitat, thereby giving generalists the capability to have a larger host range and/or colonize several habitats.

### 4.3. Speciation and Diversification in *Thaparocleidus*

Several monophyletic groups, each of them including *Thaparocleidus* species parasitizing a single host species, were observed on the basis of molecular phylogenetic reconstruction. This strongly suggests the diversification of these monogeneans by within-host speciation. For example, the parasite species of *P. nasutus* are sister species resulting from several intrahost speciation events. In contrast with *P. nasutus,* which is restricted to the Borneo and Sumatra basins [29], *P. micronema* is largely distributed in Southeast Asia. In this host, *Thaparocleidus* species belong to three different phylogenetic lineages. Among these, two lineages (the first includes the species of group 1 and the next includes the species within group 3 in Figure 3) are formed by sister species which were the result of intrahost speciation. As the different *Thaparocleidus* lineages found in *P. micronema* are not closely related to each other, multiple colonization events are likely to have occurred within this host species. *Thaparocleidus* speciation has probably occurred independently in different host species, and different *Thaparocleidus* lineages could evolve in parallel within the same host. 

The concept of sympatric speciation as an evolutionary diversification process remains controversial. According to Coyne [3] (see also [67]), there are four main requirements needed to prove sympatric speciation. The first is that the species must be largely or completely sympatric. In our case, if we consider the host species as a unit, all parasite species found within the same given host species are considered to be sympatric species. Secondly, these sympatric species must show reproductive isolation. It was demonstrated in other monogenean groups that congeneric species found in the same host species and occupying adjacent niches within the host (i.e., gill parts) differ in the morphology of their copulatory organs [68]. Thirdly, the sympatric species must be sister species, which is shown in our phylogenetic analyses where most *Thaparocleidus* species from a single host species form a monophyletic group. Fourthly and finally, the species did not seem to have undergone an allopatric diversification phase. However, a more intensive survey and analysis of the *Thaparocleidus* species will be needed to justify this assumption.

Sympatric speciation can usually be encountered when closely related species live in isolated island-like habitats. Host species are considered as islands for parasites, and in view of the parasite life cycle we can expect parasite speciation to occur at a higher rate than host speciation. This faster pace of evolution also favours intrahost speciation. Sympatric speciation in monogeneans has previously been observed in *Dactylogyrus* species parasitizing cyprinid fish in Central Europe [7]. In this system, the authors suggested that parasite diversification can be explained by sympatric speciation events (i.e., intrahost speciation). Intrahost or sympatric speciation is linked to reproductive isolation of sympatric parasite populations. Different mechanisms have been proposed to explain the reproductive isolation of parasites such as habitat selection (preferred niches are its consequence) or mate choice [22]. On the basis of tree-based cophylogenetic analysis using different event cost schemes, sympatric speciation (i.e., intrahost speciation) also appears as the dominant coevolutionary event involved in *Thaparocleidus* diversification. However, our study also evidenced some host switches in the *Thaparocleidus*-Pangasiidae system. Giraud et al. [69] showed that certain pathogen life traits (i.e., production of numerous propagules, gene exchange occurring within hosts, linkage of traits experiencing selection, and strong selection imposed by the hosts) likely render them prone to rapid ecological speciation by host shifts (i.e., speciation by specialization onto a novel host). As many such life traits have been recognized also for monogenean fish parasites, this may explain the evidence of host switches documented by cophylogenetic analyses in monogenean parasites. 

The overall congruence between the *Thaparocleidus* and Pangasiidae phylogenies was statistically significant according to topology-based and distance-based methods. Using a tree-based method, a nonsignificant global fit between the phylogenies of *Thaparocleidus* parasites and pangasiid hosts was found only using the model with a higher cost for duplication than for host switch. When considering the fact that duplication is the most numerous coevolutionary event in congeneric monogeneans parasitizing freshwater fish hosts (e.g., [7, 21]), duplication is probably not so costly as host switch is (because many monogenean species are host specific; thus, they have a limited ability for dispersal to other host species). Therefore, the model with the cost of 2 for duplication and 1 for host switch seems to be less realistic. 

While our results indicate the congruence between *Thaparocleidus* and Pangasiidae phylogenies, these results should be interpreted carefully. First, the number of the investigated host species could be small to detect potential cospeciation events even though we included all principal and commonly occurring pangasiid species from Indonesian islands. In addition to the tree-based and distance-based methods, the estimates of divergence times in host and parasite lineages are considered as critical components of cophylogenetic studies to detect cospeciation [10]. However, no timing information is available for our analysed model. Huyse and Volckaert [19] on the basis of tree-based methods found an overall fit between the phylogenies of *Gyrodactylus* parasites (viviparous monogeneans) and goby hosts, but an absolute timing of speciation events in hosts and parasites ruled out the possibility of synchronous speciation. Thus, they proposed that phylogenetically conserved host switching mimics the phylogenetic signature of cospeciation. 

Bentz et al. [70] studied the evolution of African *Polystoma*, endoparasitic monogeneans of neobatrachian hosts, and proposed that distinctive larval behaviour of polystomes engenders isolation between parasite populations which precludes sympatric speciations, and thus cospeciation is another factor of diversification of *Polystoma* in the African continent. However, the majority of previous cophylogenetic studies on congeneric monogeneans parasitizing fish did not report cospeciation [7, 17, 18, 21]. De Vienne et al. [71] in their review study showed that convincing cases of cospeciation in host-parasite and host-mutualist associations are very rare and host switches may be the dominant mode of speciation over cospeciation. In addition, they suggested that cophylogenetic methods overestimate the occurrence of cospeciation. Different processes may generate apparent cospeciation [9, 71]. Our study indicates that such apparent cospeciation in *Thaparocleidus*-Pangasiidae may be generated by intrahost duplications and/or also caused by host-switching events. The sympatric occurrence of some pangasiid species may more likely support the evidence of host switches than cospeciation in *Thaparocleidus* diversification; for instance, *P. djambal* and *P. polyuranodon* live in the same basin, which could facilitate host switching. 

## 5. Conclusion 

Our study of closely related parasites within a relatively small geographical area emphasizes particularly that intrahost speciation is the dominant coevolutionary event in *Thaparocleidus* species diversification favored by high specificity. Our study may indicate that host switches rather than cospeciation play a more substantial role in *Thaparocleidus* diversification. However, Pangasiidae speciation is closely related to tectonic events and the variation of sea levels [31, 44]; we then expected a similar pattern in parasite evolution. Therefore, to infer a formal conclusion on the role of cospeciation and host switching for *Thaparocleidus* diversification, we need to study these monogenean species on a broader geographical scale also including additional host species. Our study indicates that the morphological variability of attachment organ in *Thaparocleidus* parasites is not inherited from a common ancestor and could be potentially under adaptive constraint.

## Figures and Tables

**Table 1:** List of parasite species including their specificity (S: specialist, G: generalist), host species from which the parasite species was sequenced, and localities of collection and accession number. Host specificity was delimited using published records (see Section 2.)

Fish species	*Thaparocleidus *species	Location	Accession number
*Pangasius nasutus *	*T. caecus 1 *(G)	Borneo	FJ493153
	*T. alatus 2 *(S)	Batang Hari River (Sumatra)	FJ493156
	*T. citreum *	Musi River (Sumatra)	FJ493145
	*T. alatus 1 *(S)	Musi River (Sumatra)	FJ493146

*Pangasius micronema *	*T. durandi 1 *(S)	Borneo	FJ493151
	*T. durandi 2 *(S)	Batang Hari River (Sumatra)	FJ493162
	*T. rukyanii 2 *(S)	Batang Hari River (Sumatra)	FJ493163
	*T. tacitus *(S)	Batang Hari River (Sumatra)	FJ493161
	*T. lebrunae *(G)	Batang Hari River (Sumatra)	FJ493165
	*T. summagracilis 2 *(S)	Batang Hari River (Sumatra)	FJ493164
	*T. sinepinae *(G)	Musi River (Sumatra)	FJ493147
	*T. rukyanii 1 *(S)	Musi River (Sumatra)	FJ493148
	*T. summagracilis 1 *(S)	Musi River (Sumatra)	FJ493149

*Pangasius djambal *	*T. komarudini *(G)	Batang Hari River (Sumatra)	FJ493154
	*T. combesi *(G)	Batang Hari River (Sumatra)	FJ493155

*Pangasius polyuranodon *	*T. crassipenis *(S)	Batang Hari River (Sumatra)	FJ493157
	*T. levangi *(S)	Batang Hari River (Sumatra)	FJ493158
	*T. legendrei *(S)	Batang Hari River (Sumatra)	FJ493160
	*T. turbinatio *(G)	Batang Hari River (Sumatra)	FJ493159

*Pangasianodon hypophthalmus*	*T. caecus 2 *(G)	Borneo	FJ493152
	*T. siamensis *(S)	Borneo	FJ493150

**Table 2:** Results of cophylogenetic analyses calculated in Jane 4 for *Thaparocleidus* parasites and Pangasiidae fish. The numbers of each event type necessary to reconcile host and parasite trees under different event cost schemes are shown. Event costs in the second column correspond to the following events: cospeciation, duplication, host switch, sorting event, and failure to diverge. The significant *P* values are shown in bold.

Model	Event costs	Total cost	Cospeciation	Duplication	Duplication & host switch	Sorting event	Failure to diverge	*P* value
Jane default model v. 4	1 1 1 1 1	26	4	11	5	1	0	**0.002**
Jane default model v. 3	0 1 1 2 1	23	4	11	5	1	0	**0.016**
TreeMap default model	0 1 1 1 1	22	4	11	5	1	0	**0.045**
TreeMap default model for building a jungle	0 2 1 1 1	37	5	10	5	2	0	0.270
TreeFitter default model	0 0 2 1 1	11	4	11	5	2	0	**0.005**
Host switch-adjusted TreeFitter model	0 0 1 1 1	6	4	11	5	1	0	**0.001**
Codivergence adjusted TreeFitter model	1 0 1 1 1	8	0	12	8	0	0	**0.003**

Note: the event cost schemes including cost for each evolutionary event are shown in the second column. Because it is assumed that host switch can only occur with duplication event, Jane 4 (unlike Jane 3, TreeMap, and TreeFitter) defined “duplication and host switch” instead of “host switch” with the default cost equal to 2 (i.e., cost of 1 for duplication and 1 for host switch is equivalent in Jane 4 to a cost of 1 for duplication and 2 for “duplication and host switch”). To avoid the misinterpretation of event cost schemes used in this study, in this table we retained the presentation using the classically applied event costs (i.e., cost for duplication and cost for host switch).

## References

[1] Coyne JA, Orr HA (1998). The evolutionary genetics of speciation. *Philosophical Transactions of the Royal Society B*.

[2] Via S (2001). Sympatric speciation in animals: the ugly duckling grows up. *Trends in Ecology and Evolution*.

[3] Coyne JA (2007). Sympatric speciation. *Current Biology*.

[4] Brooks DR, McLennan DA (1991). *Phylogeny, Ecology and Behavior: A Research Program in Comparative Biology*.

[5] McCoy KD (2003). Sympatric speciation in parasites—what is sympatry?. *Trends in Parasitology*.

[6] Kunz W (2002). When is a parasite species a species?. *Trends in Parasitology*.

[7] Šimková A, Morand S, Jobet E, Gelnar M, Verneau O (2004). Molecular phylogeny of congeneric monogenean parasites (*Dactylogyrus*): a case of intrahost speciation. *Evolution*.

[8] Page RDM (2003). *Tangled Trees: Phylogeny, Cospeciation and Coevolution*.

[9] de Vienne DM, Giraud T, Shykoff JA (2007). When can host shifts produce congruent host and parasite phylogenies? A simulation approach. *Journal of Evolutionary Biology*.

[10] Light JE, Hafner MS (2008). Codivergence in heteromyid rodents (Rodentia: Heteromyidae) and their sucking lice of the genus *Fahrenholzia* (Phthiraptera: Anoplura). *Systematic Biology*.

[11] Humphery-Smith I (1989). The evolution of phylogenetic specificity among parasitic organisms. *Parasitology Today*.

[12] Hafner MS, Nadler SA (1988). Phylogenetic trees support the coevolution of parasites and their hosts. *Nature*.

[13] Hafner MS, Sudman PD, Villablanca FX, Spradling TA, Demastes JW, Nadler SA (1994). Disparate rates of molecular evolution in cospeciating hosts and parasites. *Science*.

[14] Roberts LS, Javony JJ (1996). *Foundations of Parasitology*.

[15] Poulin R (1992). Determinants of host-specificity in parasites of freshwater fishes. *International Journal for Parasitology*.

[16] Šimková A, Desdevises Y, Gelnar M, Morand S (2000). Co-existence of nine gill ectoparasites (*Dactylogyrus*: Monogenea) parasitising the roach (*Rutilus rutilus* L.): history and present ecology. *International Journal for Parasitology*.

[17] Desdevises Y, Morand S, Jousson O, Legendre P (2002). Coevolution between *Lamellodiscus* (Monogenea: Diplectanidae) and Sparidae (Teleostei): the study of a complex host-parasite system. *Evolution*.

[18] Zietara MS, Lumme J (2002). Speciation by host switch and adaptive radiation in a fish parasite genus *Gyrodactylus* (Monogenea, Gyrodactylidae). *Evolution*.

[19] Huyse T, Volckaert FAM (2005). Comparing host and parasite phylogenies: *Gyrodactylus* flatworms jumping from goby to goby. *Systematic Biology*.

[20] Plaisance L, Littlewood DTJ, Olson PD, Morand S (2005). Molecular phylogeny of gill monogeneans (Platyhelminthes, Monogenea, Dactylogyridae) and colonization of Indo-West Pacific butterflyfish hosts (Perciformes, Chaetodontidae). *Zoologica Scripta*.

[21] Mendlová M, Desdevises Y, Civáňová K, Pariselle A, Šimková A (2012). Monogeneans of West African cichlid fish: evolution and cophylogenetic interactions. *PloS ONE*.

[22] Rohde K (1979). Critical evaluation of intrinsic and extrinsic factors responsible for niche restriction in parasites. *The American Naturalist*.

[23] Morand S, Šimková A, Matějusová I, Plaisance L, Verneau O, Desdevises Y (2002). Investigating patterns may reveal processes: evolutionary ecology of ectoparasitic monogeneans. *International Journal for Parasitology*.

[24] Šimková A, Pečínková M, Řehulková E, Vyskočilová M, Ondračková M (2007). *Dactylogyrus* species parasitizing European *Barbus* species: morphometric and molecular variability. *Parasitology*.

[25] Kaci-Chaouch T, Verneau O, Desdevises Y (2008). Host specificity is linked to intraspecific variability in the genus *Lamellodiscus* (Monogenea). *Parasitology*.

[26] Lim LHS, Timofeeva TA, Gibson DI (2001). Dactylogyridean monogeneans of the siluriform fishes of the Old World. *Systematic Parasitology*.

[27] Wu X-Y, Zhu X-Q, Xie M-Q, Wang JQ, Li A-X (2008). The radiation of *Thaparocleidus* (Monogenoidea: Dactylogyridae: Ancylodiscoidinae): phylogenetic analyses and taxonomic implications inferred from ribosomal DNA sequences. *Parasitology Research*.

[28] Pariselle A, Lim LHS, Lambert A (2006). Monogeneans from Pangasiidae (Siluriformes) in Southeast Asia: X. Six new species of *Thaparocleidus* Jain, 1952 (Ancylodiscoididae) from *Pangasius micronema*. *Parasite*.

[29] Roberts TR, Vidthayanon C (1991). Systematic revision of the Asian catfish family Pangasiidae, with biological observations and descriptions of three new species. *Proceedings of the Academy of Natural Sciences of Philadelphia*.

[30] Pouyaud L, Teugels GG, Gustiano R, Legendre M (2000). Contribution to the phylogeny of pangasiid catfishes based on allozymes and mitochondrial DNA. *Journal of Fish Biology*.

[31] Voris HK (2000). Maps of Pleistocene sea levels in Southeast Asia: shorelines, river systems and time durations. *Journal of Biogeography*.

[32] Lim LHS (1990). *Silurodiscoides* Gussev, 1961 (Monogenea: Ancyrocephalidae) from *Pangasius sutchi* Fowler, 1931 (Pangasiidae) cultured in Penninsular Malaysia. *Raffles Bulletin of Zoology*.

[33] Pariselle A, Lim LHS, Lambert A (2002). Monogeneans from Pangasiidae (Siluriformes) in Southeast Asia: III. Five new species of *Thaparocleidus* Jain, 1952 (Ancylodiscoididae) from *Pangasius bocourti*, *P. djambal* and *P. hypophthalmus*. *Parasite*.

[34] Pariselle A, Lim LHS, Lambert A (2003). Monogeneans from Pangasiidae (Siluriformes) in Southeast Asia: V. Five new species of *Thaparocleidus* Jain, 1952 (Ancylodiscoididae) from *Pangasius nasutus*. *Parasite*.

[35] Pariselle A, Lim LHS, Lambert A (2004). Monogeneans from pangasiidae (Siluriformes) in Southeast Asia: VII. Six new host-specific species of *Thaparocleidus* Jain, 1952 (Ancylodiscoididae) from *Pangasius polyuranodon*. *Parasite*.

[36] Pariselle A, Lim LHS, Lambert A (2005). Monogeneans from Pangasiidae (Siluriformes) in Southeast Asia: VIII. Four new non-specific species of *Thaparocleidus* Jain, 1952 (Ancylodiscoididae) from *Pangasius polyuranodon* and *P. elongatus*. *Parasite*.

[37] Pariselle A, Lim LHS, Lambert A (2005). Monogeneans from Pangasiidae (Siluriformes) in Southeast Asia: IX. Two new species of *Thaparocleidus* Jain, 1952 (Ancylodiscoididae) from *Pangasius mahakamensis*. *Parasite*.

[38] Euzet L, Combes C, Boquet C, Genermont J, Lamotte M (1980). Les problèmes de l’espèce chez les animaux parasites. *Les Problèmes de l’espèce dans le Règne Animal*.

[39] Sinnappah ND, Lim LHS, Rohde K, Tinsley R, Combes C, Verneau O (2001). A paedomorphic parasite associated with a neotenic amphibian host: phylogenetic evidence suggests a revised systematic position for Sphyranuridae within anuran and turtle Polystomatoineans. *Molecular Phylogenetics and Evolution*.

[40] Šimková A, Plaisance L, Matějusová I, Morand S, Verneau O (2003). Phylogenetic relationships of the Dactylogyridae Bychowsky, 1933 (Monogenea: Dactylogyridea): the need for the systematic revision of the Ancyrocephalinae Bychowsky, 1937. *Systematic Parasitology*.

[41] Thompson JD, Higgins DG, Gibson TJ (1994). CLUSTAL W: improving the sensitivity of progressive multiple sequence alignment through sequence weighting, position-specific gap penalties and weight matrix choice. *Nucleic Acids Research*.

[42] Hall TA (1999). BioEdit: a user-friendly biological sequence alignment editor and analysis program for Windows 95/98/NT. *Nucleic Acids Research*.

[43] Swofford DL (2002). *PAUP*: Phylogenetic Analysis Using Parsimony and Other Methods*.

[44] Ronquist F, Huelsenbeck JP (2003). MrBayes 3: Bayesian phylogenetic inference under mixed models. *Bioinformatics*.

[45] Posada D, Crandall KA (1998). Modeltest: testing the model of DNA substitution. *Bioinformatics*.

[46] Rzhetsky A, Nei M (1992). A simple method for estimating and testing minimum-evolution trees. *Molecular Biology and Evolution*.

[47] Felsenstein J (1985). Confidence limits on phylogenies: an approach using the bootstrap. *Evolution*.

[48] Wahlberg N, Weingartner E, Nylin S (2003). Towards a better understanding of the higher systematics of Nymphalidae (Lepidoptera: Papilionoidea). *Molecular Phylogenetics and Evolution*.

[49] Yang J, He S, Freyhof J, Witte K, Liu H (2006). The phylogenetic relationships of the Gobioninae (Teleostei: Cyprinidae) inferred from mitochondrial cytochrome b gene sequences. *Hydrobiologia*.

[50] Kumar S, Tamura K, Nei M (2004). MEGA3: integrated software for molecular evolutionary genetics analysis and sequence alignment. *Briefings in Bioinformatics*.

[51] Abascal F, Zardoya R, Posada D (2005). ProtTest: selection of best-fit models of protein evolution. *Bioinformatics*.

[52] Guindon S, Gascuel O (2003). A simple, fast, and accurate algorithm to estimate large phylogenies by maximum likelihood. *Systematic Biology*.

[53] Legendre P, Desdevises Y, Bazin E (2002). A statistical test for host-parasite coevolution. *Systematic Biology*.

[54] Meier-Kolthoff JP, Auch AF, Huson DH, Göker M (2007). CopyCat: cophylogenetic analysis tool. *Bioinformatics*.

[55] Conow C, Fielder D, Ovadia Y, Libeskind-Hadas R (2010). Jane: a new tool for the cophylogeny reconstruction problem. *Algorithms for Molecular Biology*.

[56] Page RDM (1994). Parallel phylogenies: reconstructing the history of host-parasite assemblages. *Cladistics*.

[57] Ronquist F TreeFitter, program and documentation. http://www.softpedia.com/progMoreBy/Publisher-Fredrik-Ronquist-Maxim-Teslenko83341.html.

[58] Douady CJ, Delsuc F, Boucher Y, Doolittle WF, Douzery EJP (2003). Comparison of bayesian and maximum likelihood bootstrap measures of phylogenetic reliability. *Molecular Biology and Evolution*.

[59] Pol D, Siddall ME (2001). Biases in maximum likelihood and parsimony: a simulation approach to a 10-taxon case. *Cladistics*.

[60] Gusev AV (1985). *Identification of Freshwater Fish Parasites*.

[61] Pouyaud L, Desmarais E, Deveney M, Pariselle A (2006). Phylogenetic relationships among monogenean gill parasites (Dactylogyridea, Ancyrocephalidae) infesting tilapiine hosts (Cichlidae): systematic and evolutionary implications. *Molecular Phylogenetics and Evolution*.

[62] Wu X-Y, Zhu X-Q, Xie M-Q, Li A-X (2007). The evaluation for generic-level monophyly of Ancyrocephalinae (Monogenea, Dactylogyridae) using ribosomal DNA sequence data. *Molecular Phylogenetics and Evolution*.

[63] Blasco-Costa I, Míguez-Lozano R, Sarabeev V, Balbuena JA (2012). Molecular phylogeny of species of *Ligophorus* (Monogenea: Dactylogyridae) and their affinities within the Dactylogyridae. *Parasitology International*.

[64] Vignon M, Pariselle A, Vanhove MPM (2011). Modularity in attachment organs of African *Cichlidogyrus* (Platyhelminthes: Monogenea: Ancyrocephalidae) reflects phylogeny rather than host specificity or geographic distribution. *Biological Journal of the Linnean Society*.

[65] Šimková A, Verneau O, Gelnar M, Morand S (2006). Specificity and specialization of congeneric monogeneans parasitizing cyprinid fish. *Evolution*.

[66] Jarkovský J, Morand S, Šimková A, Gelnar M (2004). Reproductive barriers between congeneric monogenean parasites (*Dactylogyrus*: Monogenea): attachment apparatus morphology or copulatory organ incompatibility?. *Parasitology Research*.

[67] Morand S, Šimková A, Gourbière S (2008). Beyond the paradigms of cospeciation and host-switch: is sympatric speciation an important mode of speciation for parasites?. *Life and Environment*.

[68] Šimková A, Ondračková M, Gelnar M, Morand S (2002). Morphology and coexistence of congeneric ectoparasite species: reinforcement of reproductive isolation?. *Biological Journal of the Linnean Society*.

[69] Giraud T, Gladieux P, Gavrilets S (2010). Linking the emergence of fungal plant diseases with ecological speciation. *Trends in Ecology and Evolution*.

[70] Bentz S, Leroy S, Du Preez L, Mariaux J, Vaucher C, Verneau O (2001). Origin and evolution of African *Polystoma* (Monogenea: Polystomatidae) assessed by molecular methods. *International Journal for Parasitology*.

[71] de Vienne DM, Refrégier G, López-Villavicencio M, Tellier A, Hood ME, Giraud T (2013). Cospeciation versus host-shift speciation: methods for testing, evidence from natural associations and relation to coevolution. *New Phytologist*.

